# Supplementary material for: Gasdermin-D-dependent IL-1α release from microglia promotes protective immunity during chronic Toxoplasma gondii infection
Source: Nat Commun. 2020 Jul 23;11:3687. doi: 10.1038/s41467-020-17491-z (PMC7378823; doi:10.1038/s41467-020-17491-z)
Supplement: Supplementary file 1 — Supplementary Information [file 41467_2020_17491_MOESM1_ESM.pdf]

Gasdermin-D-dependent IL-1α release from microglia promotes protective immunity during chronic *Toxoplasma gondii* infection

Batista et al.

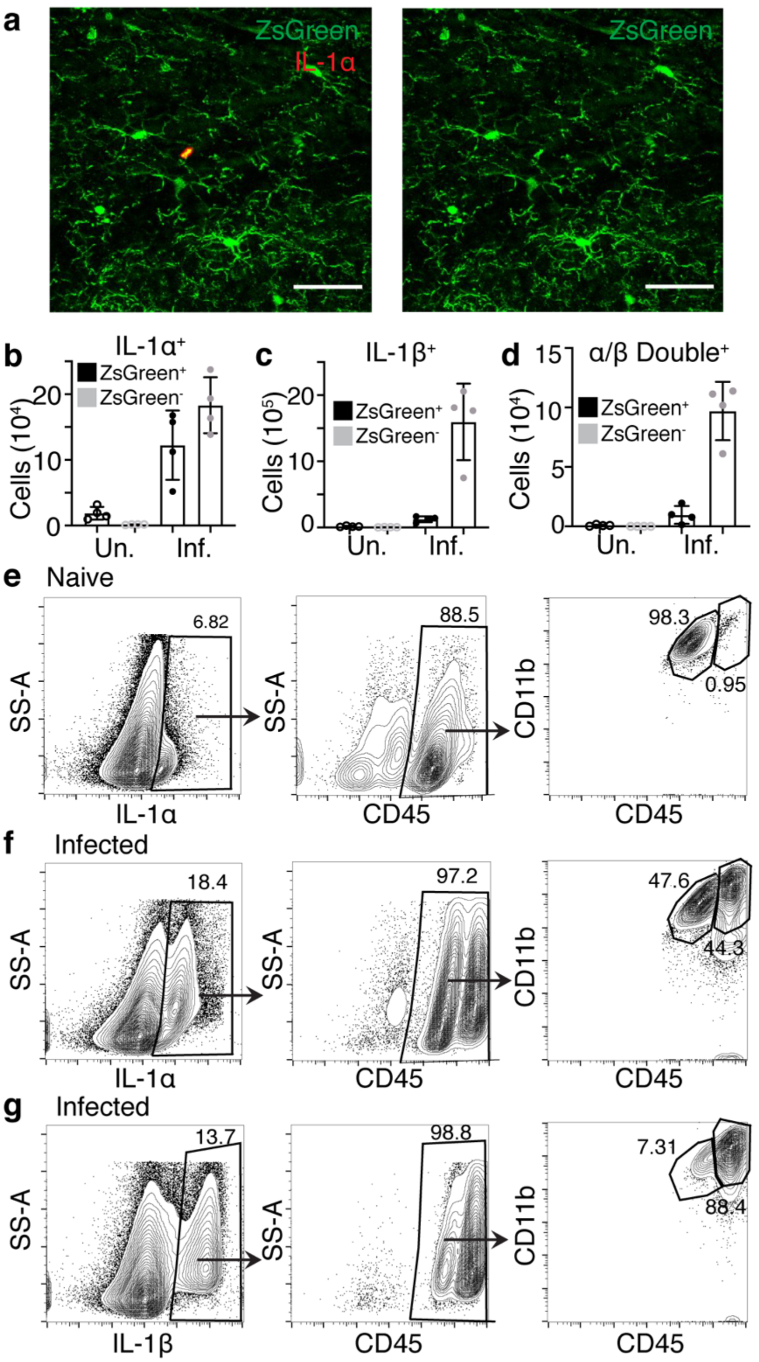

Supplementary Figure 1 | Microglia and macrophages in the infected brain differ in IL-1 expression. a-d, $CX_3CR_1^{Cre\text{-}ERT2}$ x $ZsGreen^{fl/stop/fl}$ mice were left naïve or infected with 10 cysts of Me49 strain *T. gondii* parasites for 4 weeks. (n = 4 mice per group) **a,** Representative image of IL-1α in naïve brain colocalizing with microglia, scale bar is 50 μm. **b-d,** Brains were harvested and analyzed by flow cytometry with intracellular cytokine staining. Data are presented as mean values +/- SEM. Numbers of IL-1α$^+$ (**b**), IL-1β$^+$ (**c**), and double positive (**d**) cells were quantified in both ZsGreen$^+$ and ZsGreen$^-$ populations in naïve and infected mice. Cells were pre-gated on singlets/live/ZsGreen. **e-g,** Brains from naïve or chronically infected mice were analyzed by flow cytometry. **e,** Representative plots of IL-1α expression for naïve samples, previously gated on live/singlets. **f-g,** Representative plots of IL-1α (**f**) and IL-1β (**g**) expression for infected samples.

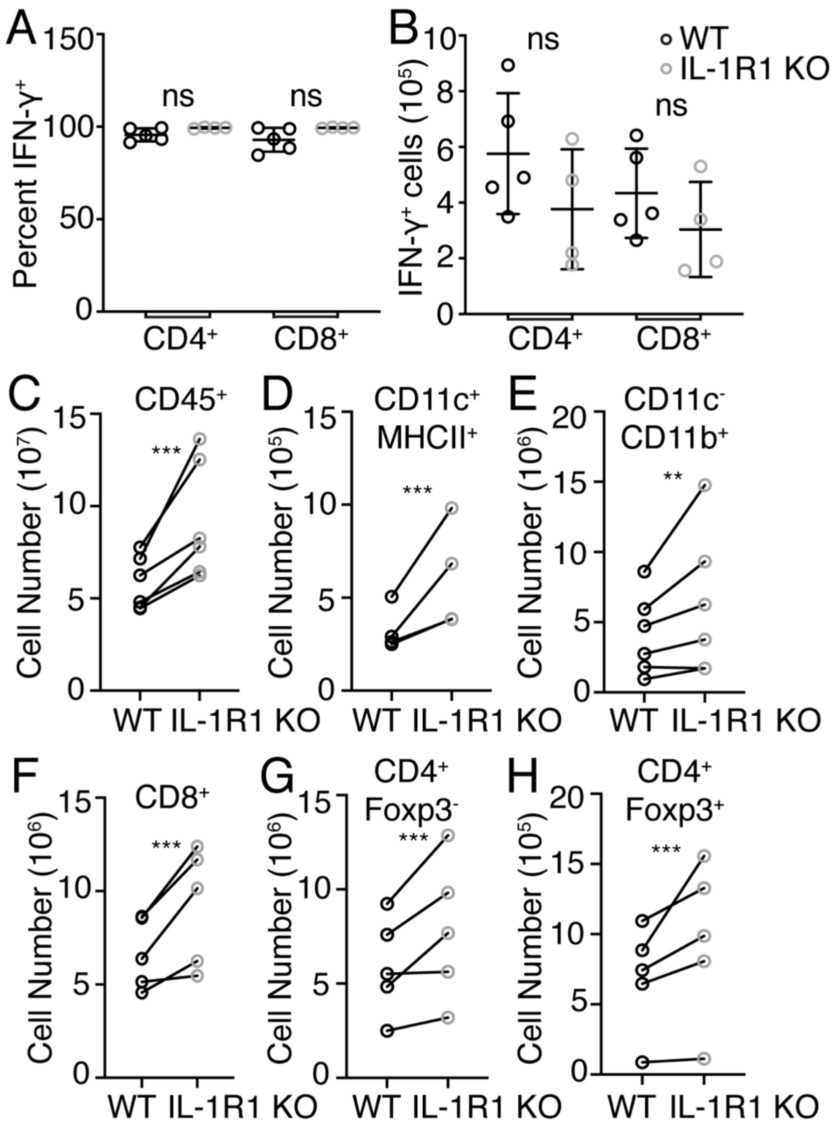

Supplementary Figure 2 | Brain IFN-γ responses and peripheral immune responses are not impaired in IL-1R1 KO mice during chronic *T. gondii* infection. WT and IL-1R1 KO mice were infected i.p. with 10 cysts of the Me49 strain of *T. gondii*. 6 weeks p.i. brains (**a-b)** and spleens (**c-h**) were harvested and processed for flow cytometry. Immune cell populations were enumerated. **a-b,** Brains were harvested and digested. Isolated cells were incubated at 37°C for 5 hours with a mix of PMA/ionomycin and brefeldin A. Intracellular cytokine staining was performed and analyzed by flow cytometry. Cells were pre-gated on singlets/Live/$CD3^+$ and percent (**a**) and number (**b**) of IFN-γ^+ CD4 and $CD8^+$ T cells were determined. **c,** Total immune cells, pre-gated on singlets/live, $p = 2.64 \times 10^{-6}$ **d,** DCs, pre-gated on singlets/live/$CD45^+$/$Dump^-$(CD3/NK1.1/B220), $p = 6.19 \times 10^{-4}$ **e,** Monocytes/ macrophages, pre-gated on singlets/live/ $CD45^+$/$CD11c^-$/$CD11b^+$/$CD45^{hi}$, $p = 0.001$ **f,** $CD8^+$ T cells, pre-gated on singlets/live/$CD3^+$, $p = 1.39 \times 10^{-4}$ **g,** Effector $CD4^+$ T cells, pre-gated on singlets/live/$CD3^+$, $p = 5.52 \times 10^{-4}$ **h,** Tregs, pre-gated on singlets/live/$CD3^+$, $p = 9.63 \times 10^{-4}$ **a-b,** A representative experiment is shown and statistics were performed using a two-tailed Student's T test. (n = 9 mice) Data are presented as mean values +/- SEM. **c-h,** Paired averages compiled from 3-6 experiments. Statistics were performed using a randomized block ANOVA. (n = 49 mice)

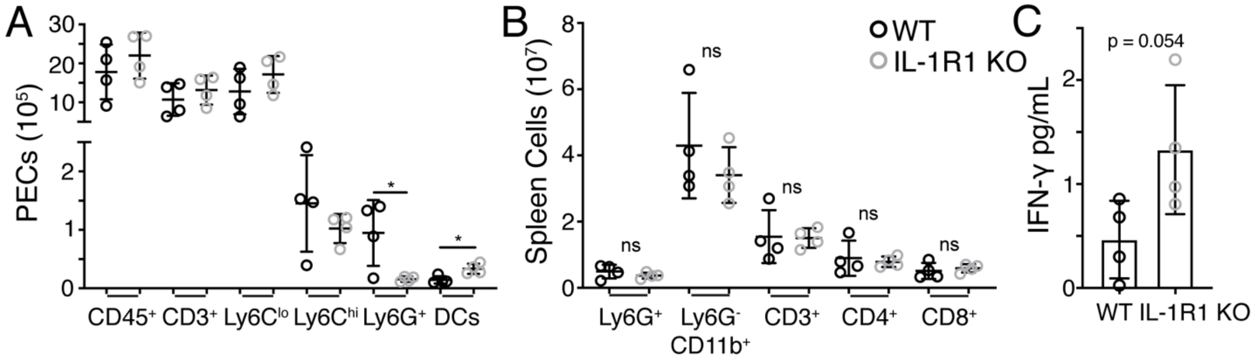

Supplementary Figure 3 | IFN-γ response and monocyte/macrophage response during acute infection are not impaired in IL-1R1 KO mice. WT and IL-1R1 KO mice were infected i.p. with 10 cysts of the Me49 strain of *T. gondii*. Mice were sacrificed 12 days p.i. **a,** Peritoneal lavage was performed and peritoneal exudate cells (PECs) were isolated and analyzed by flow cytometry. For Ly6G$^+$ cells $p = 0.03$, for DCs $p = 0.01$. **b,** Spleen cells were isolated and analyzed by flow cytometry. **c,** Serum was harvested at the time of sacrifice and IFN-γ in the serum was analyzed by ELISA. A representative experiment is shown (n = 4 mice per group). Statistics were performed using a two-tailed Student's t-test between groups for each measure. Data are presented as mean values +/- SEM.

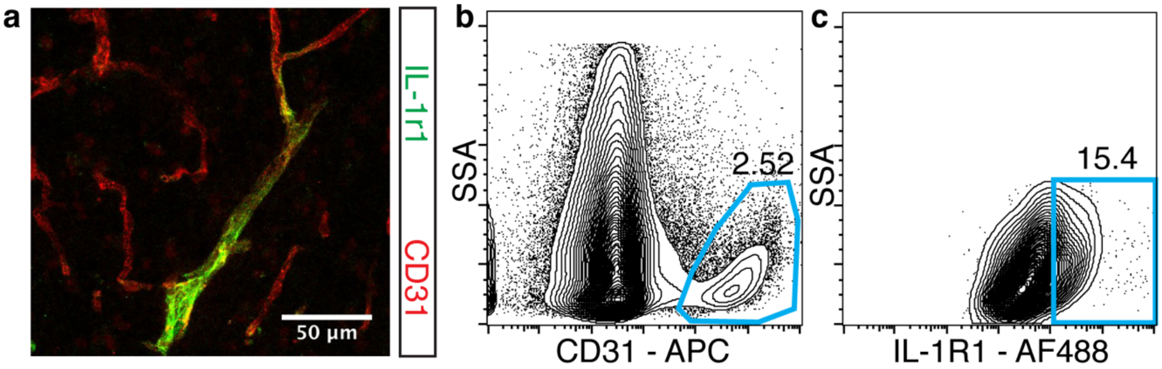

Supplementary Figure 4 | IL-1R1 is expressed by endothelial cells in the brain. a, Brains from chronically infected C57B6/J mice were harvested, fixed, and stained with antibodies against CD31 (red) and IL-1R1 (green). Representative of two independent experiments. **b-c,** Brains from uninfected C57B6/J mice were harvested and processed for flow cytometry analysis. Cells were previously gated on Singlets/Live/CD45$^-$ and then were gated on CD31$^+$ (**b**) and IL-1R1 (**c**) expression on the CD31$^+$ population.

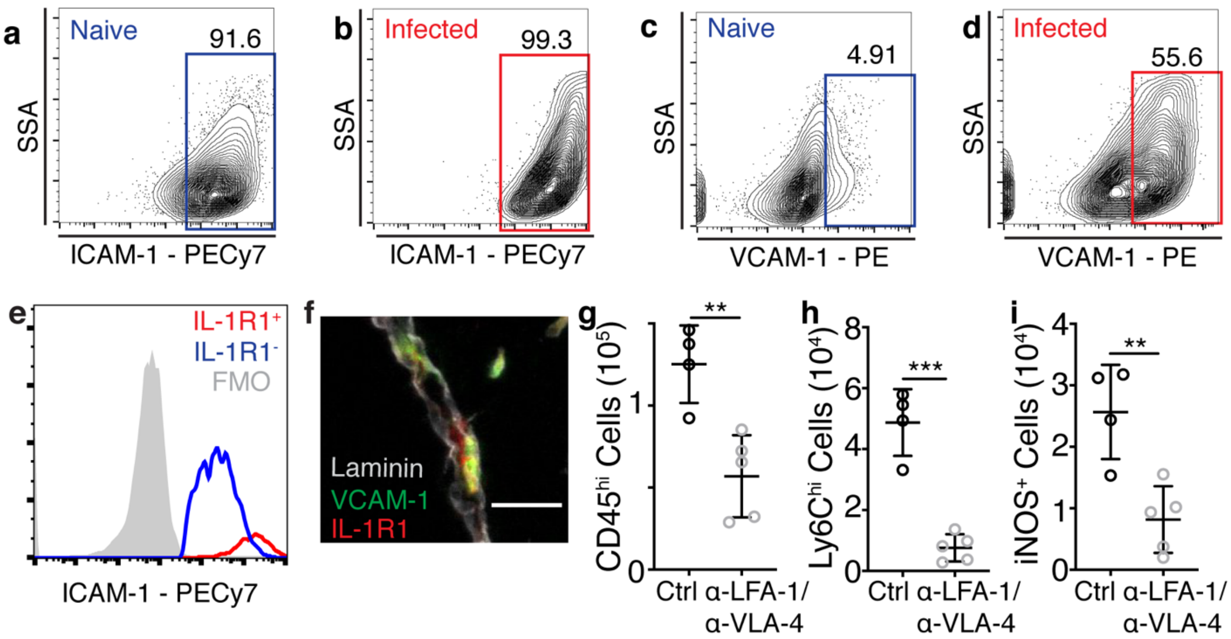

Supplementary Figure 5 | The brain endothelium is activated during chronic *T. gondii* infection. a-d, WT C57B6/J mice were either left naïve or infected i.p. with the Me49 strain of *T. gondii*. 4 weeks p.i. mice were sacrificed and brains were harvested for flow cytometry analysis. (n = 2 mice per group) **a-b,** Samples were pre-gated on singlets/live/Hoescht^{+}/CD45^{-}/CD31^{+} and then ICAM-1 expression was assessed. Representative plots from naïve (**a**) and infected (**b**) mice are shown. **c-d,** Samples were pre-gated as in **a** and then VCAM-1 expression was assessed. Representative plots from naïve (**c**) and infected (**d**) mice are shown. **e,** Histogram showing ICAM-1 expression on IL-1R1 positive and negative endothelial cells, the FMO is shown in filled gray **f,** Brains from chronically infected C57B6/J mice were harvested, fixed, and stained with antibodies against laminin (gray), IL-1R1 (red), and VCAM-1 (green). Scale bar = 50 μm **g-i,** C57B6/J mice were infected i.p. with 10 cysts of the Me49 strain of *T. gondii*. 4 weeks p.i. mice were treated with either control IgG or 200 μg each of α-LFA-1 and α-VLA-4 blocking antibodies on days 1 and 3 of treatment, and were sacrificed on day 5. Brains were harvested and processed for flow cytometry. (n = 9 mice) **g,** Cells were previously gated on singlets/live/CD11c^{-}/CD45^{+} and the numbers of CD11b^{+}CD45^{hi} cells are shown. $p = 0.004$. Of the CD45^{hi} cells numbers of Ly6C^{hi} cells, $p = 0.0001$ (**h**) and iNOS^{+} cells, $p = 0.005$ (**i**) were enumerated. Statistics were performed using a two-tailed Student's T-test. Data are presented as mean values +/- SEM.

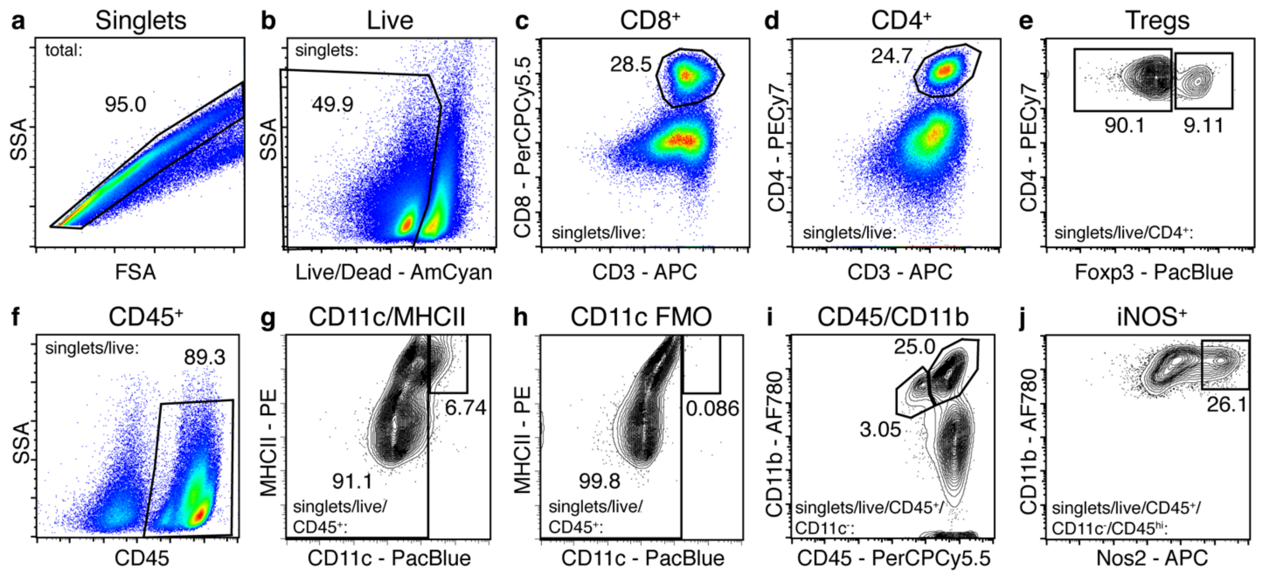

Supplementary Figure 6 | Example gating strategy for brain immune populations. Myeloid and T cell populations were identified using two separate panels. **a-b,** for all panels, samples were first gated on singlets and then cells which excluded the live/dead dye. **c-e,** to identify T cell populations, live cells were plotted to gate on either CD8+CD3+ (**c**) or CD4+CD3+ (**d**) cells. **e,** to identify Tregs, CD4+ T cells were gated on Foxp3+. **f-j,** to identify myeloid cell populations live cells were first gated on CD45+ (**f**). CD45+ cells were then gated on CD11c and MHCII (**g**), CD11c+MHCII hi cells were called DCs. CD11c- cells were then gated by CD45 and CD11b (**i**). CD11b+CD45int cells were called microglia, and CD11b+CD45hi cells were called infiltrating myeloid cells. CD45hi cells were then gated on iNOS+ (**j**).
