# Supplementary material for: Gasdermin-D-dependent IL-1α release from microglia promotes protective immunity during chronic Toxoplasma gondii infection
Source: Nat Commun. 2020 Jul 23;11:3687. doi: 10.1038/s41467-020-17491-z (PMC7378823; doi:10.1038/s41467-020-17491-z)
Supplement: Supplementary file 3 — Reporting Summary [file 41467_2020_17491_MOESM3_ESM.pdf]

nature research

Corresponding author(s): Tajie Harris

Last updated by author(s): Jun 4, 2020

Reporting Summary

Nature Research wishes to improve the reproducibility of the work that we publish. This form provides structure for consistency and transparency in reporting. For further information on Nature Research policies, see our Editorial Policies and the Editorial Policy Checklist.

Statistics

For all statistical analyses, confirm that the following items are present in the figure legend, table legend, main text, or Methods section.

n/a	Confirmed	
☐	☒	The exact sample size (*n*) for each experimental group/condition, given as a discrete number and unit of measurement
☐	☒	A statement on whether measurements were taken from distinct samples or whether the same sample was measured repeatedly
☐	☒	The statistical test(s) used AND whether they are one- or two-sided<br>*Only common tests should be described solely by name; describe more complex techniques in the Methods section.*
☐	☒	A description of all covariates tested
☐	☒	A description of any assumptions or corrections, such as tests of normality and adjustment for multiple comparisons
☐	☒	A full description of the statistical parameters including central tendency (e.g. means) or other basic estimates (e.g. regression coefficient) AND variation (e.g. standard deviation) or associated estimates of uncertainty (e.g. confidence intervals)
☐	☒	For null hypothesis testing, the test statistic (e.g. *F*, *t*, *r*) with confidence intervals, effect sizes, degrees of freedom and *P* value noted<br>*Give P values as exact values whenever suitable.*
☒	☐	For Bayesian analysis, information on the choice of priors and Markov chain Monte Carlo settings
☐	☒	For hierarchical and complex designs, identification of the appropriate level for tests and full reporting of outcomes
☒	☐	Estimates of effect sizes (e.g. Cohen's *d*, Pearson's *r*), indicating how they were calculated

Our web collection on statistics for biologists contains articles on many of the points above.

Software and code

Policy information about availability of computer code

Data collection	No code was used in data collection. Kaluza for Gallios was used in collection of flow cytometry data. Metamorph and Leica acquisition software were used in collection of microscopy data.
Data analysis	Prism 8, R v.3.4.4, FlowJo v.10.6.1, FastQC v0.11.5, R Bioconductor package DESeq2 (v.1.16.1); Enhanced Volcano (v.1.2.0); AnnotationDbi (v.1.46.0); clusterProfiler (v.3.12.0); pheatmap package (v1.0.12); TrimmomaticPE: 0.39 -threads 6 -phred33 LEADING:3 TRAILING:3 SLIDINGWINDOW:4:15 MINLEN:36; Salmon (v.0.8.2); numBootstraps 30; Mouse genome version: gencode_mouse_m13; Tximport (v.1.4.0); Pypiper (v.0.6.0), python (v.2.7.14)

For manuscripts utilizing custom algorithms or software that are central to the research but not yet described in published literature, software must be made available to editors and reviewers. We strongly encourage code deposition in a community repository (e.g. GitHub). See the Nature Research guidelines for submitting code & software for further information.

Data

Policy information about availability of data

All manuscripts must include a data availability statement. This statement should provide the following information, where applicable:

- Accession codes, unique identifiers, or web links for publicly available datasets
- A list of figures that have associated raw data
- A description of any restrictions on data availability

The sequencing data that support the findings of this study are available under the GEO accession number GSE146680. The source data underlying Figs 2a-e, 4d-k, 6e-i, and Supplementary Fig 2c-h are provided as a Source Data file.

Field-specific reporting

Please select the one below that is the best fit for your research. If you are not sure, read the appropriate sections before making your selection.

- [x] Life sciences
- [ ] Behavioural & social sciences
- [ ] Ecological, evolutionary & environmental sciences

For a reference copy of the document with all sections, see nature.com/documents/nr-reporting-summary-flat.pdf

Life sciences study design

All studies must disclose on these points even when the disclosure is negative.

Sample size	Pilot studies and power analysis determined that quantification of immune cell populations by flow cytometry requires 6 animals to achieve a power of 0.8. Where possible, 6 animals per group was the goal, though controlled groups often were not able to reach 6. Thus, we combined experiments statistically using a Randomized Block ANOVA to correct for any effect of experiment date.
Data exclusions	No data were excluded from the analysis.
Replication	All major phenotypes were replicated at least 2-3 times (different animals and different infections). Trends were consistent across experiments.
Randomization	For experiments using naive and infected wild-type mice, different cages of mice were randomly assigned to groups. For experiments involving treatment with chemical inhibitors on ex vivo samples, wells were randomly assigned to treatment and samples deriving from different animals were equally represented in each treatment group. For experiments involving genetic knockout mice, mice groups were defined by genotype.
Blinding	The investigator was blinded to sample identity during counting of parasite cysts, which was determined to be the measure most likely to be affected by unintended bias. The investigator was not blinded during tissue harvest or processing, but all samples were handled in the same way. The investigator was not blinded during acquisition or analysis of flow cytometry data, but the same gates were applied to all samples in a given experiment.

Reporting for specific materials, systems and methods

We require information from authors about some types of materials, experimental systems and methods used in many studies. Here, indicate whether each material, system or method listed is relevant to your study. If you are not sure if a list item applies to your research, read the appropriate section before selecting a response.

Materials & experimental systems

n/a	Involved in the study	
[ ]	[x]	Antibodies
[x]	[ ]	Eukaryotic cell lines
[x]	[ ]	Palaeontology and archaeology
[ ]	[x]	Animals and other organisms
[x]	[ ]	Human research participants
[x]	[ ]	Clinical data
[x]	[ ]	Dual use research of concern

Methods

n/a	Involved in the study	
[x]	[ ]	ChIP-seq
[ ]	[x]	Flow cytometry
[x]	[ ]	MRI-based neuroimaging

Antibodies

Antibodies used	For flow cytometry: CD31 (Invitrogen, clone 390, ref 17-0311-80, lot 1994125), CD45 (eBioscience, clone 30-F11, ref 35-0451-80, lot E16146-104), MHCII (BD, clone M5/114.15.2, Cat 557000, lot 7264993), NK1.1 (eBioscience, clone PK136, Cat 11-5941-85, lot E00743-1630), CD19 (Invitrogen, clone 1D3, ref 11-0193-85, lot 4329189), CD3 (Invitrogen, clone 17A2, ref 50-0032-82, lot 1965660), CD4 (Invitrogen, clone GK1.5, ref 25-0041-82, lot 1993630), CD8a (Invitrogen, clone 53-6.7, ref 45-0081-82, lot 2017819), CD11c (Invitrogen, clone N418, ref 45-0114-82, lot 1933266), CD11b (eBioscience, clone M1/70, ref 47-0112-82, lot 4290718), Foxp3 (Invitrogen, clone FJK-16s, ref 48-5773-82, lot 4332193), Ly6G (BioLegend, clone 1A8, Cat 127612, lot B251507), Ly6C (Invitrogen, clone HK1.4, ref 25-5932-82, lot 1990189), IFN-y (eBioscience, clone XMG1.2, Cat 17-7311-82, lot E028188), Nos2 (Invitrogen, clone CXNFT, ref 17-5920-80, lot 1990771), IL-1a (eBioscience, clone ALF-161, ref 12-7011-81, lot E02048-1635), pro-IL-1b (eBioscience, clone NJTEN3, ref 25-7114-82, lot E18008-103).<br>For IHC: pro-IL-1b (same as above), iNOS (Invitrogen, Prod PA5-16855, lot SD2370711L), CD31 (Invitrogen, clone 390, ref 14-0311-82, lot 4349120), VCAM-1 (BioLegend, clone 429, Cat 105702, lot B217557), ICAM-1 (eBioscience, clone YN1/1.7.4, ref 13-0541-82, lot 4284903), c-Rel (Santa Cruz Biotechnology, clone G-7, cat sc-365720, lot E1517), IL-1a (R&D Systems, clone ALF-161, Cat AF-400-NA, lot AMG0214111), IL-1R1 (R&D Systems, Cat AF771, lot EVJ0115041), p65 (Cell signalling technology, clone D14E12, ref 8242S, lot 13), Iba1 (Wako, Cat 019-19741), Laminin (Cedarlane Labs, prod CL54851AP-1).

	For Ab blockade: anti-LFA-1 (BioXCell, clone M17/4, cat BE0006), anti-VLA-4 (BioXCell, clone PS/2, cat BE0071)
Validation	All antibodies were validated by the manufacturer.<br>For flow antibodies:<br>CD31: Species reactivity, mouse. Usage, flow (flow). CD45: Species reactivity, mouse. Usage, flow. MHCII: Species reactivity, mouse. Usage, flow. NK1.1: Species reactivity, mouse. Usage, flow. CD19: Species reactivity, mouse. Usage, flow. CD3: Species reactivity, mouse. Usage, flow, immunocytochemistry (ICC), immunohistochemistry (IHC), immunofluorescence (IF). CD4: verified by relative expression. Species reactivity, mouse. Usage, flow. CD8a: Species reactivity, mouse. Usage, flow. CD11c: Species reactivity, mouse. Usage, flow. CD11b: Species reactivity, mouse. Usage, flow. Foxp3: verified by relative expression. Species reactivity, Bovine Dog, Cat, Mouse, Pig, Rat. Usage, flow. Ly6G: Ly6G transfected EL-4J cell line. Species reactivity, mouse. Usage, flow. Ly6C: Species reactivity, mouse. Usage, flow. IFN-γ: Verified by cell treatment. Species reactivity, mouse. Usage, flow. Nos2: Species reactivity, mouse. Usage, flow. IL-1a: Species reactivity, mouse. Usage, flow. Pro-IL-1b: Species reactivity, mouse. Usage, flow.<br>For IHC antibodies:<br>Pro-IL-1b: (same as above). iNOS: Verified by cell treatment. Species reactivity, Human, Mouse, Rat. Usage, ICC, IHC, IF, Western Blot (WB). CD31: Species reactivity, mouse. Usage, flow, IHC, immunoprecipitation (IP). VCAM-1: Mouse preadipose cell line PA6. Species reactivity, mouse. Usage, flow. ICAM-1: Species reactivity, mouse. Usage, flow. c-Rel: Species reactivity, mouse, rat, human. Usage, WB, IP, IF, ELISA. IL-1a: E. coli-derived recombinant mouse IL-1alpha. Species reactivity, mouse. Usage, WB, IHC. IL-1R1: Mouse myeloma cell line NS0-derived recombinant mouse IL-1R1. Species reactivity, mouse. Usage, WB, IHC. p65: Species reactivity, human, mouse, rat, hamster, monkey, dog. Usage, flow, WB, IHC, IF, IP, CHIP. Iba1: Species reactivity, human, mouse, rat. Usage, ICC. Laminin: Species reactivity, mouse, rat. Usage, IF, IHC, ELISA.<br>For blocking antibodies:<br>Anti-LFA-1: C57BL/6 mouse splenic secondary cytotoxic T cells. Species reactivity, mouse. Usage, in vivo neutralization, flow. Anti-VLA-4: Mouse P815 mast cells. Species reactivity, mouse. Usage, in vivo neutralization, in vitro neutralization, flow.

Animals and other organisms

Policy information about studies involving animals; ARRIVE guidelines recommended for reporting animal research

Laboratory animals	Laboratory mice were used in this study. Strains used include: WT (C57B6/J) (CD45.2),WT (C57B6/J) CD45.1), IL-1R1 KO, IL-1a KO, IL-1b KO, Caspase-1/11 KO, Gasdermin-D KO, CX3CR1cre-ERT2 x ZsGreen fl/stop/fl, ASC-citrine, and CX3CR1cre-ERT2 x ZsGreen fl/stop/fl X ASC citrine. Mice were infected between the ages of 7-8 weeks. Both males and females were used. In all experiments, groups were age- and sex-matched.
Wild animals	The study did not involve wild animals.
Field-collected samples	The study did not involve field-collected samples.
Ethics oversight	Animal protocol was approved by the Institutional Animal Care and Use Committee at the University of Virginia.

Note that full information on the approval of the study protocol must also be provided in the manuscript.

Flow Cytometry

Plots

Confirm that:

- [x] The axis labels state the marker and fluorochrome used (e.g. CD4-FITC).
- [x] The axis scales are clearly visible. Include numbers along axes only for bottom left plot of group (a 'group' is an analysis of identical markers).
- [x] All plots are contour plots with outliers or pseudocolor plots.
- [x] A numerical value for number of cells or percentage (with statistics) is provided.

Methodology

Sample preparation	Tissue processing: Immediately after sacrifice mice were perfused with 30 mL of cold 1X PBS. Brains and spleens were harvested and put into cold complete RPMI media (cRPMI) (10% FBS, 1% penicillin/streptomycin, 1% sodium pyruvate, 1% non-essential amino acids, and 0.1% 2-ME). If peritoneal lavage fluid was collected, prior to perfusion, 5 mL of cold 1X PBS was injected through the intact peritoneal membrane with a 26-gauge needle, and removed with a 22-gauge needle. If serum was collected, blood from the heart was collected and allowed to clot at 4C overnight to separate serum.<br>After harvest, brains were minced with a razor blade, passed through an 18-gauge needle, and then enzymatically digested with 0.227 mg/mL collagenase/dispase and 50 U/mL DNase (Roche) at 37C for 45 minutes. After digestion, brains homogenate was passed through a 70m filter (Corning) and washed with cRPMI. To remove myelin from samples, filtered brain homogenate was then resuspended with 20 mL of 40% Percoll and spun at 650 x g for 25 minutes. Myelin was aspirated, samples were washed with cRPMI, and then resuspended in cRPMI. Spleens were mechanically homogenized and passed through a 40 um filter (Corning). Samples were washed with cRPMI and then resuspended in 2 mL of RBC lysis buffer (0.16 M NH4Cl) for 2 minutes. Cells were then washed with cRPMI and then resuspended. Peritoneal lavage fluid was washed with cRPMI, pelleted and resuspended.<br>Flow Cytometry and cell sorting: Single cell suspensions from tissue samples were plated in a 96-well U-bottom plate. Cells were initially incubated with 50 uL Fc block (1 ug/mL 2.4G2 Ab (BioXCell), 0.1% rat gamma globulin (Jackson ImmunoResearch)) for 10 minutes at room temperature. Cells were then surface stained with antibodies and a Live/Dead stain for 30 minutes at 4 degrees C. After surface staining, cells were washed with FACS buffer (0.2% BSA and 2 mM EDTA in

	1X PBS) and fixed at 4 degrees C for 30 minutes with either 2% paraformaldehyde (PFA) or a fixation/permeabilization kit (eBioscience). Cells were then permeabilized and stained with any intracellular markers for 30 minutes at 4 degrees C. Samples were then washed, resuspended in FACS buffer. For cell sorting, CX3CR1creERT2 x ZsGreenfl/stop/fl mice were used. After surface staining, live cells were analyzed.
Instrument	Beckman Coulter Gallios Flow Cytometer. For cell sorting BD FACSAria Fusion cell sorter was used.
Software	Kaluza for Gallios was used for data acquisition. FlowJo (v.10.6.1) was used for data analysis.
Cell population abundance	For cell sorts, ZsGreen+ population averaged about 5% of total events and the ZsGreen- population averaged about 20% of total events. Conflict events were discarded, leading to sorting efficiency of about 75%.
Gating strategy	Gating strategies are indicated in figure legends and depicted in Supplementary figure 6.<br>Figure 1: singlets/live/CD45+/CD11b+/ZsGreen+ and -<br>Figure 2 myeloid cells: singlets/live/CD45+/CD11c-/CD11b+/CD45hi/iNOS+<br>Figure 2 T cells: singlets/live/CD3+/CD4+ or CD8+<br>Figure 4 myeloid cells: singlets/live/CD45+/CD11c-/CD11b+/CD45hi/iNOS+<br>Figure 4 T cells: singlets/live/CD3+/CD4+ or CD8+<br>Figure 5: singlets/live/CD45+/CD11b+/ZsGreen+ and -<br>Figure 6 myeloid cells: singlets/live/CD45+/CD11c-/CD11b+/CD45hi/iNOS+<br>Figure 6 T cells: singlets/live/CD3+/CD4+ or CD8+<br>Figure 7 DCs: singlets/live/CD45+/CD11c+MHCIIhi<br>Figure 7 other myeloid: singlets/live/CD45+/CD11c-/CD11b+/CD45hi/iNOS+<br>Figure 7 T cells: singlets/live/CD3+/CD4+ or CD8+

☒ Tick this box to confirm that a figure exemplifying the gating strategy is provided in the Supplementary Information.
